# DenVar: density-based variation analysis of multiplex imaging data

**DOI:** 10.1093/bioadv/vbac039

**Published:** 2022-05-23

**Authors:** Souvik Seal, Thao Vu, Tusharkanti Ghosh, Julia Wrobel, Debashis Ghosh

**Affiliations:** Department of Biostatistics and Informatics, University of Colorado CU Anschutz Medical Campus, Aurora, CO, USA

## Abstract

**Summary:**

Multiplex imaging platforms have become popular for studying complex single-cell biology in the tumor microenvironment (TME) of cancer subjects. Studying the intensity of the proteins that regulate important cell-functions becomes extremely crucial for subject-specific assessment of risks. The conventional approach requires selection of two thresholds, one to define the cells of the TME as positive or negative for a particular protein, and the other to classify the subjects based on the proportion of the positive cells. We present a threshold-free approach in which distance between a pair of subjects is computed based on the probability density of the protein in their TMEs. The distance matrix can either be used to classify the subjects into meaningful groups or can directly be used in a kernel machine regression framework for testing association with clinical outcomes. The method gets rid of the subjectivity bias of the thresholding-based approach, enabling easier but interpretable analysis. We analyze a lung cancer dataset, finding the difference in the density of protein HLA-DR to be significantly associated with the overall survival and a triple-negative breast cancer dataset, analyzing the effects of multiple proteins on survival and recurrence. The reliability of our method is demonstrated through extensive simulation studies.

**Availability and implementation:**

The associated *R* package can be found here, https://github.com/sealx017/DenVar.

**Supplementary information:**

Supplementary data are available at *Bioinformatics Advances* online.

## 1 Introduction

In recent years, various technologies are being used for probing single-cell spatial biology, for example, multiparameter immunofluorescence (Bataille *et al.*, 2006), imaging mass cytometry (Ali *et al.*, 2020), multiplex immunohistochemistry (mIHC) (Tan *et al.*, 2020; Vu *et al.*, 2021) and multiplexed ion beam imaging (MIBI) (Angelo *et al.*, 2014; Seal *et al.*, 2021). These technologies, often referred to as multiplex tissue imaging, offer the potential for researchers to explore the basis of many different biological mechanisms. Multiplex tissue imaging platforms such as Vectra 3.0 (Akoya Biosciences) (Huang *et al.*, 2013), Vectra Polaris (Akoya Biosciences) (Pollan *et al.*, 2020) and MIBI (Ionpath Inc.) (Angelo *et al.*, 2014) produce images with similar structure. In particular, each image is two-dimensional, collected at cell- and nucleus-level resolution and proteins in the sample are labeled with antibodies that attach to cell membranes. We will refer to the antibodies as markers in the paper. Typically, mIHC images have 6–8 markers, whereas MIBI images can have more than 40 markers.

The majority of the above markers are surface or phenotypic markers (Shipkova and Wieland, 2012) which are primarily used for cell type identification. Additionally, there are several functional markers including HLA-DR (Saraiva *et al.*, 2018), PD1, PD-L1 and Lag3 (Phillips *et al.*, 2021) that dictate or regulate important cell-functions. Both surface and functional markers are quantified as continuous-valued marker intensities. For a phenotypic marker, a threshold is drawn to indicate whether a cell is positive or negative for the particular marker. Then one or more of these binarized phenotypic markers are used to classify the cells into different types based on biological knowledge of marker expression pattern. With functional markers, the interest lies in finding out if over-expression of the markers across the cells of the tumor microenvironment (TME) (Binnewies *et al.*, 2018) have significant impact on subject-level clinical outcomes, such as survival or recurrence (Johnson *et al.*, 2020; Koguchi *et al.*, 2015). A two-step thresholding-based approach (e.g. Bulian *et al.*, 2014; Costa *et al.*, 2017) with one marker at a time is typically used in this context which we describe next.

The two steps in the thresholding-based approach involve identifying cells positive for a functional marker and classifying subjects into different groups according to the proportions of positive cells. The group labels can be used in a linear regression framework to test association with the outcomes of interest (Chang *et al.*, 2018; Yang *et al.*, 2019). For example, Johnson *et al.* (2021) define the cells to be positive for HLA-DR (also known as, MHCII) if the corresponding mean marker intensity is >0.05. Next, they classify the subjects into two groups, MHCII: High and MHCII: Low if the proportion of cancer cells positive for HLA-DR is greater or smaller than 5%, respectively. Finally, they test if these two groups of subjects have different 5-year overall survival. Instead of grouping the subjects based on the proportion of positive cells, another approach is to directly test if the vector of the proportion of positive cells is associated with the outcome (Patwa *et al.*, 2021).

The aforementioned thresholding-based method clearly requires judicious selection of the cutoffs that greatly influence the subsequent steps of the analysis (Harris *et al.*, 2022). The result is bound to vary for different thresholding values; and a poor choice of thresholds may produce an uninformative and uninterpretable result. There is a plethora of helpful guidelines for choosing these thresholds in different contexts (Cossarizza *et al.*, 2019; Kimball *et al.*, 2018). However, there is no universal solution or rule of thumb. Thus, the method remains prone to subjectivity bias and lacks robustness. In addition, discarding important marker information by binarizing them (Altman and Royston, 2006) can be critical in capturing subtle differences between the subjects and thus, result in a loss of power and robustness (refer to the Supplementary Figs S3 and S4).

In this article, we propose a threshold-free method for distinguishing the difference between the subjects with respect to the distribution of a functional marker in the TME. We treat the expression of a marker as a continuous random variable having realizations in different cells of the TME of a subject.  Then, we compare the marker’s probability distribution or equivalently, probability density across all the subjects. Our algorithm is as follows. First, for every subject, the probability density of the marker is estimated using kernel density estimation (KDE) (Silverman, 2018). Next, a density based distance (Basu *et al.*, 1998) known as Jensen–Shannon distance (JSD) (Endres and Schindelin, 2003; Nielsen, 2019) is used to quantify the difference in the estimated density between the pairs of subjects. The matrix of distances between the subjects can then be used to classify them into meaningful groups using hierarchical clustering (Murtagh and Legendre, 2014), and the group-labels can be tested for association with clinical outcomes in a linear regression framework. Alternatively, the distance matrix can also be used directly in a linear mixed model (Hoffman, 2013; Seal *et al.*, 2022) or equivalently, a kernel machine regression framework (Jensen *et al.*, 2019; Liu *et al.*, 2008) to test for association with clinical outcomes.

Using our proposed method, we analyzed an mIHC dataset on lung cancer (Johnson *et al.*, 2021) from the University of Colorado School of Medicine, finding that the difference in HLA-DR marker density in tumor cells is associated with 5-year overall survival probability of subjects. We have also applied the proposed method on a publicly available triple negative breast cancer (TNBC) dataset (Keren *et al.*, 2018) from the MIBI platform finding the density of an immunoregulatory protein, PD1 to have significant effect on disease recurrence probability. We have performed extensive simulation studies mimicking the characteristics of the real datasets to check the power, reliability and robustness of our method.

## 2 Materials and methods

Suppose there are *M* functional markers and *N* subjects with the *j*-th subject having *n_j_* cells. Let *X_kij_* denote the scaled expression, between 0 and 1, of marker *k* in *i*-th cell of subject *j* for $k=1,2,\ldots ,M,\;i=1,2,\ldots ,n_{j}$, and j=1,2,…,N. Let *Y* (N×1 vector) be a subject-level outcome of interest and *C* be an *N *×* p* matrix of *p* subject-level covariates. Next, we describe the traditional and proposed methods, considering one marker at a time.

### 2.1 Traditional thresholding-based approach for clustering subjects

To study if abundance of marker *k* is associated with a subject’s survival or any other outcome of interest (*Y*), the conventional approach is to classify the subjects into two or more groups using a thresholding-based approach. First, we choose a threshold *t*_1_. Then we compute the number of cells for subject *j* whose expression is greater than *t*_1_ i.e., the number of cells with $X_{\mathrm{kij}}>t_{1}.$ Such cells are referred to as the cells positive for marker *k*. The proportion of the cells positive for a marker *k* in subject *j* is denoted as, $p_{kj}=\sum_{i=1}^{n_{j}}I(X_{\mathrm{kij}}>t_{1})/n_{j}$, where I(.) is the indicator function. The next threshold *t*_2_ is chosen to classify the subjects into two groups. This is based on $p_{kj}>t_{2}$ or not. Then, we test for association between the group label and clinical outcomes. This can easily be extended to allow more than two groups.

Denote the clustering variable as $Z_{kj}\equiv I(p_{kj}>t_{2})$. When *Y* is a continuous outcome, a standard multiple linear regression model with $Z_{k}={\left(Z_{k1},\ldots ,Z_{kN}\right)}^{T}$ as a predictor can be written as

$$Y=C\beta +Z_{k}\gamma_{k}+\epsilon ,$$

where β,γ are fixed effects and *ϵ* is an N×1 error vector following multivariate normal distribution (MVN) with mean **0** and identity covariance matrix $\sigma^{2}I_{N}.$ After estimating the parameters, the null hypothesis, $H_{0}:\gamma_{k}=0$, can be tested using the Wald test (Gourieroux *et al.*, 1982).

Next, we consider the case of *Y* being a survival or recurrence outcome. Let the outcome of the *j*-th individual be $Y_{j}=\min(T_{j},U_{j})$, where *T_j_* is the time to event and *U_j_* is the censoring time. Let $\delta_{j}\equiv I(T_{j}\leq U_{j})$ be the corresponding censoring indicator. Assuming that *T_j_* and *U_j_* are conditionally independent given the covariates for j=1,2,…,N, the hazard function for the Cox proportional hazards (PH) model (Andersen and Gill, 1982) with fixed effects can be written as,

(1) $$\lambda_{j}(t|C_{j},Z_{kj})=\lambda_{0}(t)\;\text{exp}\;(C_{j}^{T}\beta +Z_{kj}\gamma_{k}),\;j=1,2,\ldots ,N.$$

In Equation 1, $\lambda_{j}(t|C_{j},Z_{kj})$ is the hazard of the *j*-th subject at time *t*, given the vector of covariates *C_j_* and the cluster label *Z_kj_* and $\lambda_{0}(t)$ is an unspecified baseline hazard at time *t*. To test the null hypothesis: $H_{0}:\gamma_{k}=0$, a likelihood ratio test (LRT) (Therneau, 1997) can be considered.

As pointed out earlier, the biggest difficulty with this approach lies in choosing the thresholds, *t*_1_ and *t*_2_ appropriately. In most cases, one would perform the analysis for different pairs of (*t*_1_, *t*_2_), and choose the result that aligns best with the biological mechanism of interest. Thus, the step of threshold-selection remains entirely subjective and the results are bound to vary largely depending on the selected thresholds.

### 2.2 Proposed method: distance-based clustering using marker probability density of subjects

To avoid the bias inherent in the thresholding-based approach, we propose a distance between the subjects based on each marker *k* that would be devoid of subjectivity and can easily be tested for association with an outcome of interest. First, we discuss the concept of divergence or distance between two probability distributions and then its implementation.

#### 2.2.1 Jensen–Shannon distance

Let (X,A) be a measurable space (Billingsley, 2008) where X denotes the sample space and A the *σ*-algebra of measurable events. Consider a dominating measure *μ* and denote the set of probability distributions as P={P:A→[0,1]}. In this context, JSD (Endres and Schindelin, 2003; Nielsen, 2019) between two probability distributions, P,Q∈P can be defined as,

(2) $$d(P,Q)=\sqrt{\int_{X}p(x)\;\text{log}\;\frac{2p(x)}{p(x)+q(x)}d\mu (x)+\int_{X}q(x)\;\text{log}\;\frac{2q(x)}{p(x)+q(x)}d\mu (x)}$$

where *p*, *q* are the Radon–Nikodym derivatives or densities (Nikodym, 1930) of *P* and *Q* with respect to a dominating measure μ. Unlike other divergences between distributions, such as Kullback-Leibler divergence (van Erven and Harremos, 2014), the JSD satisfies the properties of being a metric (Lawvere, 1973) between probability measures. To formalize this, a metric d:P×P→[0,∞) needs to satisfy the following three axioms:

1. Identity: d(P,Q)=0 iff *P* = *Q*,
2. Symmetry: d(P,Q)=d(Q,P),
3. Triangle Inequality: d(P,Q)+d(Q,R)≥d(P,R) where R∈P.

Note that, *P *=* Q* implies p(x)=q(x) almost everywhere w.r.t *μ* (Athreya and Lahiri, 2006). JSD lies between (0, 2 log ⁡(2)), and smaller values associate with more similar distributions. As JSD satisfies the metric properties, it can readily be used to construct a valid distance matrix between random variables (rv’s) having different probability distributions or densities. The distance matrix can then be used in subsequent analysis such as classifying the rv’s into meaningful groups. JSD has been used in many different areas, such as bioinformatics (Sims *et al.*, 2009), social sciences (DeDeo *et al.*, 2013), and more recently, in generative adversarial networks (Goodfellow *et al.*, 2014), a popular technique in deep learning. Next, we discuss the formulation of JSD in our context.

For every subject *j*, we assume that the expression of marker *k* is a continuous random variable, denoted by *X_kj_*, taking values between 0 and 1. *X_kj_* is observed in *n_j_* cells as, $X_{k1j},X_{k2j},\;\ldots ,X_{kn_{j}j}$. Let the probability distribution function and the density function of *X_kj_* be denoted by, *F_kj_* and *f_kj_*, respectively. Next, we consider the set-up described with X=[0,1] and A being the corresponding *σ*-algebra of measurable events. Then the set, P contains the distribution functions, *F_kj_* for j=1,2,…,N and k=1,2,…,M. Using Equation 2, the distance between two subjects (j,j′) in terms of the probability distribution of marker *k* can be quantified by

$$\begin{array}{c} {\text{JSD}}_{\mathrm{kjj}\prime }=d(F_{kj},F_{kj\prime })\\ =\sqrt{\int_{0}^{1}f_{kj}(x)\;\text{log}\;\frac{2f_{kj}(x)}{f_{kj}(x)+f_{kj\prime }(x)}dx+\int_{0}^{1}f_{kj\prime }(x)\;\text{log}\;\frac{2f_{kj\prime }(x)}{f_{kj}(x)+f_{kj\prime }(x)}dx.} \end{array}$$

A large value of ${\text{JSD}}_{\mathrm{kjj}\prime }$ will imply that there is a clear difference in the distribution or equivalently, density of *k*-th marker between the pair of subjects, (j,j′). A small value will imply that the distributions are close. The distance matrix between all the subjects based on *k*-th marker can then be constructed as, ${\text{JSD}}_{k}=[[{\text{JSD}}_{\mathrm{kjj}\prime }]]$.

In a real-data analysis, the density function *f_kj_* will be unknown. Therefore, we compute corresponding KDE ${\hat{f}}_{kj}$ (Silverman, 2018) using the observations: *X_kij’_*s for $i=1,\ldots ,n_{j}$. ${\hat{f}}_{kj}$ typically has the form: ${\hat{f}}_{kj}(x)={\left(n_{j}\right)}^{-1}\sum_{i=1}^{n_{j}}w_{h}\left(x-X_{\mathrm{kij}}\right)$, where *w_h_* is a Gaussian kernel with bandwidth parameter *h*, chosen using Silverman’s rule of thumb (Silverman, 2018). Using the KDEs, ${\text{JSD}}_{\mathrm{kjj}\prime }$ can be estimated as,

(3) $$\begin{array}{c} {\hat{\text{JSD}}}_{\mathrm{kjj}\prime }=\sqrt{\sum_{r=1}^{R}\left[{\hat{f}}_{kj}(x_{r})\;\text{log}\;\frac{2{\hat{f}}_{kj}(x_{r})}{{\hat{f}}_{kj}(x_{r})+{\hat{f}}_{kj\prime }(x_{r})}+{\hat{f}}_{kj\prime }(x_{r})\;\text{log}\;\frac{2{\hat{f}}_{kj\prime }(x_{r})}{{\hat{f}}_{kj}(x_{r})+{\hat{f}}_{kj\prime }(x_{r})}\right]} \end{array}$$

where $x_{r},r=1,\ldots ,R$ are some grid-points in the interval [0,1]. In our simulations and real data analysis, the estimates did not change for sufficiently large values of *R*. We kept *R* at 1024 and chose equidistant grid-points. We made sure that the estimated densities integrate to 1 by appropriately scaling them.

#### 2.2.2 Using the distance in association analysis

Next, we construct suitable tests for testing the association of the distance matrix with dependent variable, *Y*.

*Test based on hierarchical clustering*: The estimated distance matrix (${\hat{\text{JSD}}}_{k}$) can be subjected to hierarchical clustering (Murtagh and Legendre, 2014) for classifying the subjects into two or more groups. Suppose, we obtain a vector of cluster labels: $Z_{k}={\left(Z_{k1},\ldots ,Z_{kN}\right)}^{T}$. Then, exactly the same models, described in Section 2.1 and the corresponding tests, can be used to determine if the differential expression of the *k*-th marker is associated with *Y*.

*Test based on linear mixed model*: The distance matrix can be transformed into a similarity matrix (Vert *et al.*, 2004) as, $G_{k}=\text{exp}(-{\hat{\text{JSD}}}_{k})$. When *Y* is a continuous outcome, *G_k_* can be incorporated in a linear mixed model framework, particularly popular in the context of heritability estimation (Hoffman, 2013; Seal *et al.*, 2022), as,

$$Y=C\beta +g_{k}+\epsilon ,$$

where β is the vector of fixed effects, $g_{k}={\left(g_{k1},g_{k2},\ldots ,g_{kn}\right)}^{T}$ is the vector of random effects following MVN($0,\sigma_{gk}^{2}G_{k}$) and *ϵ* is an error vector following MVN(**0**, $\sigma^{2}I_{N}).$ The null hypothesis: $H_{0}:\sigma_{gk}^{2}=0$ can be tested using a LRT (Crainiceanu and Ruppert, 2004). Note that, such a linear mixed model setup has been shown to be equivalent to a kernel machine regression framework by Liu *et al.* (2008). In a standard kernel machine regression framework, there is one additional width parameter, *ρ* that has to be estimated.

**Fig. 1.**
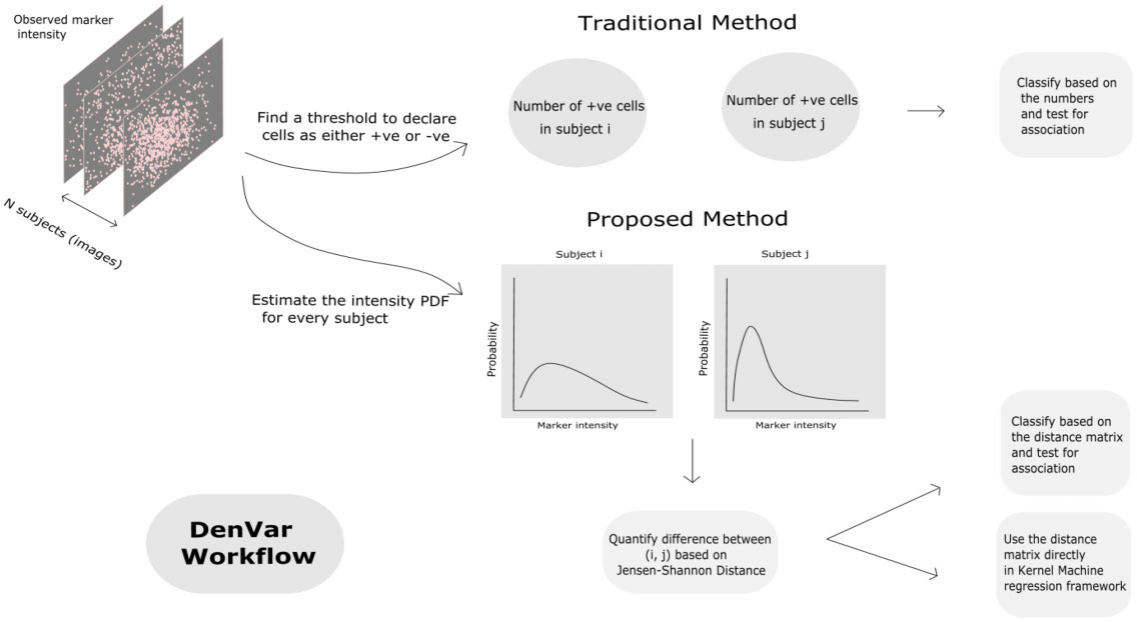
A simple comparison of the workflow of the proposed method with the traditional thresholding-based method

Next, we consider the case of *Y* being a survival or recurrence outcome. Using the same definitions and conditional independence assumptions of *T_j_*, *U_j_* and covariates as in Section 2.1, the hazard function for the Cox PH model with random effects (Therneau *et al.*, 2015) can be written as,

(4) $$\lambda_{j}(t|C_{j},g_{kj})=\lambda_{0}(t)\;\text{exp}\;(C_{j}^{T}\beta +g_{kj}),\;j=1,2,\ldots ,n$$

where $\lambda_{j}(t|C_{j},g_{kj})$ is the hazard of the *j*-th subject at time *t*, given the vector of covariates *C_j_* and the random effect *g_kj_* and $\lambda_{0}(t)$ is an unspecified baseline hazard at time *t*. To test the null hypothesis, $H_{0}:\sigma_{gk}^{2}=0$, an LRT based on integrated partial likelihoods (Therneau *et al.*, 2015) can be considered. However, it is to be kept in mind that usually a large sample size is needed to obtain a precise estimate of the random effect variance (Bell *et al.*, 2010; Maas and Hox, 2005). The problem would possibly be exacerbated in the Cox PH model with random effects because the partial likelihood would depend on the number of events (Kocak and Onar-Thomas, 2012). Therefore, we do not recommend using this test unless the sample size is sufficiently large. We have summarized the workflow of our method in Figure 1.

### 2.3 Clustering based on marker quantiles

For comparison with the traditional and proposed methods, we also consider a simpler clustering algorithm based on the subject-specific quantiles of the marker intensity. For every subject *j*, we compute a few quantiles (e.g. 95%,97.5%, 99% quantiles) of the marker intensity, i.e., $X_{\mathrm{kij}},i=1,\ldots ,n_{j}.$ Next, K-means clustering algorithm (Likas *et al.*, 2003) is used to classify the subjects into different groups based on the vector of quantiles. Once, we have the vector of cluster labels: $Z_{k}={\left(Z_{k1},\ldots ,Z_{kN}\right)}^{T}$, the hypothesis tests described in Section 2.1, can be used to determine if the differential expression of the *k*-th marker is associated with the dependent variable *Y*.

Instead of considering the entire marker density, here we are checking how well only a few quantiles of the marker distribution can capture the differences across subjects. The method can be interpreted as a less general and weaker version of the proposed JSD-based clustering. In real data, as we will see in the next section, the difference between the tails of the estimated distributions seem to be apparent and thus, the method can be expected to perform moderately well. However, choosing how many and which quantiles to use, remain subjective and dependent on careful inspection of the estimated distributions. We evaluate the performance of the method only in the simulation studies.

## 3 Real-data analysis

We first discuss the application of our method on the real datasets. We analyzed two datasets: an mIHC lung cancer dataset (Johnson *et al.*, 2021) and a MIBI breast cancer dataset (Keren *et al.*, 2018). The first dataset has a single functional marker, HLA-DR and the second dataset has four immunoregulatory proteins, PD1, PD-L1, Lag3 and IDO. We applied our proposed method on both the datasets. In all the analyses, the markers were scaled to have expression value between 0 and 1.

### 3.1 Application to mIHC lung cancer data

**Fig. 2.**
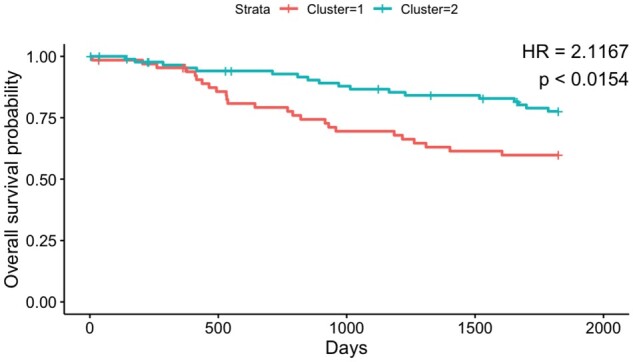
Kaplan–Meier curves for 5-year overall survival probability of 153 subjects from the lung cancer dataset, color coded by the clusters found comparing HLA-DR marker density in CK+ tumor cells. Also, displayed are the hazard ratio (HR) and the *P*-value corresponding to the test, $H_{0}:\gamma =0$ from Equation 1. Notice that HR is large (> 2) and the *P*-value is significant as well indicating that the two clusters have significant difference in survival probability

In the mIHC lung cancer dataset, there are 153 subjects each with 3–5 non-overlapping images (in total, 761 images). The subjects have varying number of cells identified (from 3755 to 16949). The cells come from two different tissue regions: tumor and stroma. The cells are pre-classified into either of the six different cell types: CD14+, CD19+, CD4+, CD8+, CK+ and Other, based on the expression of phenotypic markers, CD19, CD3, CK, CD8 and CD14. A functional marker, HLA-DR (also known as MHCII), is also measured in each of the cells. Using the thresholding-based approach described in Section 2.1, Johnson *et al.* (2021) classified the subjects into two groups, (i) MHCII: High and (ii) MHCII: Low based on the proportion of CK+ tumor cells that are also positive for HLA-DR. They discovered that there is a significant difference in 5-year overall survival between the groups. Analogously, we were interested in answering the question: whether 5-year overall survival of a subject was associated with the HLA-DR density in CK+ tumor cells. We first computed the JSD matrix between the subjects as discussed in Section 2.2.1 based on the density of HLA-DR marker in CK+ tumor cells. Next, we performed a hierarchical clustering using the computed JSD matrix to classify the subjects into two groups. Finally, we tested if there was a difference in survival probability between the subjects of the two groups using the test based on the Cox PH model described in Section 2.2.2 (and Equation 1). Figure 2 shows the Kaplan–Meier curves (Efron, 1988) of the two groups of subjects. We noticed that hazard ratio (HR) was large (>2) and the *P*-value was significant (<0.015) indicating that 5-year overall survival probability was associated with the probability density of HLA-DR in CK+ tumor cells.

**Fig. 3.**
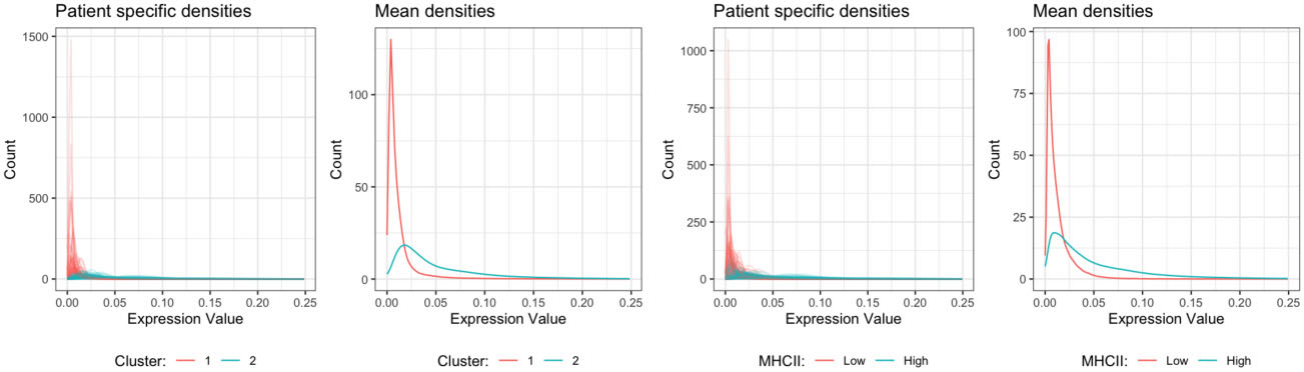
Two figures on the left respectively correspond to individual and mean HLA-DR probability density in CK+ tumor cells of the subjects (patients) from the two clusters found using the proposed JSD-based clustering. Two figures on the right respectively correspond to individual and mean HLA-DR marker probability density in CK+ tumor cells of the subjects from the two groups identified by Johnson *et al.* (2021)’s traditional thresholding-based method. Notice that the distinction between the density profiles of the identified clusters (groups) is more apparent in our method than the traditional method.

Next, we checked the degree of concordance between Johnson *et al.* (2021)’s classification and the classification based on our method and summarized it in Table 1. The accompanying values of Rand index (RI) and adjusted Rand index (ARI) were 0.64 and 0.29, respectively, indicating that the classifications moderately agreed with each other. We investigated how the estimated HLA-DR density profiles varied across the two clusters found by our method and also, across the groups identified by Johnson *et al.* (2021)’s traditional classification. From Figure 3, we noticed that the individual densities from cluster 1 were more right-skewed compared to those from Cluster 2 which led to the mean density of cluster 1 having a very high mode compared to that of Cluster 2. Some of the subjects from MHCII: High group actually had density functions similar to the mean density of MHCII: Low group meaning that the thresholding-based method was incapable of fully capturing the differences between the density profiles.

**Table 1.** Number of subjects common between the groups found using the thresholding-based method and our proposed method in the lung cancer dataset

	Cluster 1	Cluster 2
MHCII: High	80	17
MHCII: Low	18	38

We also used the test based on Cox PH model with random effects from Section 2.2.2 in this case. The estimated variance of the random effect was 0.38. Following Therneau *et al.* (2015)’s interpretation of the variance parameter in this context, we concluded that there are multiple subjects in the study with quite large relative risks, $\text{exp}(\sqrt{0.38})=1.855$ fold greater than the average subjects. However, the LRT based on integrated partial likelihoods was not significant.

### 3.2 Application to TNBC MIBI data

**Fig. 4.**
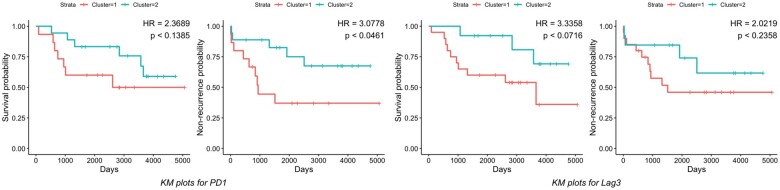
Kaplan–Meier curves for survival and recurrence probability of 33 subjects color coded by the clusters found using our method on the markers, PD1 (two figures on the left) and Lag3 (two figures on the right). Note that the difference in PD1 density has significant effect on recurrence probability

The TNBC MIBI dataset has images from 41 subjects. Keren *et al.* (2018) categorized these subjects into three groups: ‘cold’, ‘compartmentalized’ and ‘mixed’ based on the level of immune infiltration in the TME. We were interested in studying the density of the immunoregulatory protein markers, PD1, PD-L1, and Lag3 which have been shown to have immunological relevance (Keren *et al.*, 2018; Patwa *et al.*, 2021). PD1 and Lag3 are primarily expressed in immune cells and ‘cold’ subjects have very few immune cells expressing them. Thus, we focused our analysis on 33 non-‘cold’ subjects. For PD1 and Lag3, we studied their density only in immune cells of a subject and for PD-L1, we studied its density both in immune and tumor cells of a subject. For every marker, we computed the JSD matrix between the subjects and performed a hierarchical clustering to classify the subjects into two groups. Then, we tested the vector of cluster labels for association with two available outcomes: disease recurrence and survival using the Cox PH model. Figure 4 shows the Kaplan–Meier curves corresponding to the markers PD1 and Lag3 (refer to the Supplementary Material for PD-L1). We noticed that there was significant difference in recurrence probability between the clusters obtained using marker PD1 (HR = 3.0778, *P* < 0.0461). For other two markers, we did not find any statistically significant results (at level 0.05). However, it is worth pointing out that the HR corresponding to survival for the clusters obtained using Lag3 was large (HR = 3.3358, *P* < 0.0716), alluding to a possible association of Lag3 density with the risk of death. We should also keep in mind that the sample size for this particular analysis was quite low which could have limited our power.

## 4 Simulation study application

Next, we compared the performance of the proposed JSD-based clustering with the thresholding-based method and also the simpler marker quantile based method (Section 2.3) in terms of ARI (Santos and Embrechts, 2009), in three different simulation setups. In the first two setups, we simulated expression data based on the mean HLA-DR expression profiles of the two clusters found in the mIHC lung cancer dataset (Section 3.1), while in the last one, we simulated expression data using the assumptions of the thresholding-based method. In all the three setups, we considered two groups of subjects (referred to as Groups 1 and 2) with sizes $N_{1}=60$ and $N_{2}=40$. Each subject had *n* cells. And, two values of *n*, 200 and 2000 were considered.

We had noticed that the mean HLA-DR distributions of the two clusters identified in the mIHC lung cancer dataset could be well approximated using Beta distributions (Gupta and Nadarajah, 2004) with different sets of parameters (*α*, *β*). In particular, the mean distribution of the Cluster 1 could be well approximated by Beta(2.17, 300), whereas the mean distribution of the Cluster 2 could be well approximated by Beta(1.78, 45). Refer to the Supplementary Material for more details. The essential difference between these two distributions was that the former had a much sharper peak and a thinner tail compared to the latter. These distributions and their perturbations were respectively used in the following two simulation setups.

### 4.1 Simulation using mean expression profile of the Cluster 1 of the mIHC data

For a subject from the Group 1, the marker expression in every cell was simulated from Beta(2.17, 300). For a subject from the Group 2, the marker expression in every cell was simulated from Beta(*x*, 300) where *x* was chosen such that the mode of this distribution was higher than the mode of the Group 1 distribution by a percentage of *l*. Five different values of *l*, 10,20,50,100 and 200 were considered. We considered 100 replications in every case.

As discussed earlier, the thresholding-based approach requires specifying two thresholds *t*_1_ and *t*_2_. We varied *t*_1_ between 95% and 97.5% quantiles of the full marker data (concatenating marker data of all the subjects) and kept *t*_2_ at 0.01. These two methods were referred to as 95% and 97.5% thresholding respectively. For the marker quantile based method, we considered the vector of three extreme quantiles, 97.5%,99% and 99.5%. Table 2 lists the average ARI of the methods across all the replications. Refer to the Supplementary Material for a table of the confidence interval of ARI for the different methods.

**Table 2.** Performance of different methods in terms of the average ARI (higher is better) across 100 replications in the simulation setup from Section 4.1

Number of cells	% difference in modes	JSD-based clustering	95% thresholding	97.5% thresholding	Marker quantiles
*n *=* *200	10	0.0744	0.0014	0.0234	0.0052
	20	0.3988	0.0029	0.0551	0.0312
	50	0.9808	0.0236	0.2264	0.2200
	100	1.0000	0.1979	0.6324	0.6864
	200	1.0000	0.8628	0.9570	0.9876
*n *=* *2000	10	0.8029	0.0000	0.0000	0.0760
	20	0.9530	0.0000	0.0001	0.3136
	50	1.0000	0.0000	0.0299	0.9350
	100	1.0000	0.0040	0.8105	0.9996
	200	1.0000	0.9907	1.0000	1.0000

We noticed that when the number of cells and differences in modes were both small (n=200,l=10), all the methods performed poorly. The performance of the methods expectedly improved as the difference *l* increased and the JSD-based clustering achieved close to 1 accuracy even for a moderate difference in modes (*l *=* *50). For large number of cells (*n *=* *2000), the JSD-based clustering achieved 80% accuracy even for the smallest *l*, whereas the thresholding-based approaches achieved little to zero accuracy for all the smaller values of *l*. For both the values of *n*, the marker quantile based clustering performed relatively well and beat the thresholding-based approaches in most of the scenarios. It demonstrated the novelty of capturing the difference of marker distribution, even in a simpler form, across subjects to classify them into meaningful groups.

### 4.2 Simulation using mean expression profile of the Cluster 2 of the mIHC data

For a subject from the Group 1, the marker expression in every cell was simulated from Beta(1.78, 45). For a subject from the Group 2, the marker expression in every cell was simulated from Beta(*x*, 45) where *x* was chosen such that the mode of this distribution was higher than the mode of the Group 1 distribution by a percentage of *l*. Five different values of *l*, 10,20,50,100 and 200 were considered. Table 3 lists the average ARI of the methods across 100 replications. Refer to the Supplementary Material for a table of the confidence interval of ARI for the different methods.

**Table 3.** Performance of different methods in terms of the average ARI across 100 replications in the simulation setup from Section 4.2

Number of cells	% difference in modes	JSD-based clustering	95% thresholding	97.5% thresholding	Marker quantiles
*n *=* *200	10	0.0345	0.0005	0.0171	0.0025
	20	0.2003	0.0016	0.0411	0.0160
	50	0.8656	0.0119	0.1395	0.1308
	100	0.9996	0.0699	0.4153	0.4455
	200	1.0000	0.5428	0.8696	0.9196
*n *=* *2000	10	0.5157	0.0000	0.0000	0.0403
	20	0.9737	0.0000	0.0001	0.1788
	50	1.0000	0.0000	0.0045	0.7795
	100	1.0000	0.0000	0.2627	0.9925
	200	1.0000	0.4074	0.9984	1.0000

Once again, the JSD-based clustering outperformed the thresholding-based approaches in all the cases. Interestingly enough, the thresholding-based approaches seemed to be performing worse in this simulation setup compared to the previous one. Possibly, a different set of (*t*_1_, *t*_2_) would have been more appropriate in this scenario. It reiterated the point that the subjectivity of the thresholding-based approaches can hugely alter or affect the performance. The marker quantile based method performed better than the thresholding-based approaches, showing again why comparing the marker distributions across the subjects can turn out be to more informative and useful than the thresholding-based analysis.

### 4.3 Simulation under the assumptions of the thresholding-based method

Next, we devised a simulation setup where the true values of the thresholds: (*t*_1_, *t*_2_) were known and the marker expression data were generated based on that. Recall that *t*_1_ controls how one defines a cell to be positive for a marker and *t*_2_ controls how one clusters the subjects into two groups based on the number of positive cells. We again considered two groups of subjects with the subjects from Group 1 having $t_{2}\%$ positive cells and the subjects from Group 2 having more than $t_{2}\%$ positive cells. Two different values of *t*_1_, 0.05,0.1 and five different values of $t_{2},0.005,0.01,0.05,0.1$ and 0.2 were considered. Refer to the Supplementary Material for more details about the simulation strategy. Table 4 lists the average ARI of the JSD-based clustering across 100 replications for all combinations of the parameters. We found out that our method performed better for higher values of *t*_2_. The value of *t*_1_ and the value of *n* did not have any apparent impact on the performance. It should be kept in mind that using the thresholding approach in this simulation setup with the known values of (*t*_1_, *t*_2_), one would achieve ARI accuracy of 1 in all the cases. However, as we have repeatedly pointed out, knowing the true values of (*t*_1_, *t*_2_) will never be possible in real data.

**Table 4.** Performance of the JSD-based clustering in terms of the average ARI across 100 replications in the simulation setup from Section 4.3

Number of cells	$t_{2}:$	0.005	0.01	0.05	0.1	0.2
*n *=* *200	$t_{1}=0.05$	0.760	0.791	0.801	0.938	1.000
	$t_{1}=0.1$	0.784	0.727	0.815	0.957	0.987
*n *=* *2000	$t_{1}=0.05$	0.778	0.727	0.800	0.936	1.000
	$t_{1}=0.1$	0.784	0.727	0.808	0.965	1.000

## 5 Discussion

In multiplexed tissue imaging datasets, it is of interest to stratify the subjects based on the profile of a functional marker in the TME for the purpose of risk assessment (e.g. risk of recurrence and risk of death). The most common approach is a thresholding-based method which requires elaborate tuning of two or more thresholds, one to binarize the marker expression and others to group the subjects based on the binarized expression. In consequence, the method remains largely subjective and varies from one researcher to another based on their interpretation of the data. On top of that, discarding valuable marker information by binarizing can result in loss of power and robustness. In this article, we have developed a threshold-free method for classifying subjects based on the probability density of the functional markers. The method is easy to interpret and free from the subjectivity bias.

In our method, we treat the expression-profile of a functional marker in a subject as a continuous random variable and compute its kernel density estimate based on its observed expression value in the cells of the TME. Once the marker density estimates for all the subjects have been computed, we use Jensen–Shannon distance (JSD) to quantify the difference in marker densities between the subjects. If the distance between two subjects is large, it means that they have very different marker expression-profiles. Next, the computed distance matrix is used in either of the following two ways. It can be subjected to hierarchical clustering to group the subjects into clusters and the cluster-labels can be tested for association with outcomes of interest (e.g. recurrence, survival). Or, it can be used directly in a kernel machine regression setup for testing association with outcomes of interest. We briefly discuss one more simpler method which takes into account the difference of the marker distribution across subjects in terms of only a few quantiles. The marker quantile based method can be interpreted as a weaker or less general version of the proposed JSD-based method.

We analyzed two highly complex multiplex tissue imaging datasets, an mIHC lung cancer dataset from University of Colorado School of Medicine and a publicly available TNBC MIBI data. In the lung cancer dataset, we discovered that the difference in HLA-DR marker density between subjects was significantly associated with their 5-year overall survival. In the breast cancer dataset, we found out that the difference in the density of immunoregulatory protein PD1 was associated with the disease recurrence. Next, we replicated the characteristics of the lung cancer dataset in two simulation setups and showcased the robustness of our method in comparison with the thresholding-based method. Interestingly enough, even the marker-quantile based method outperformed the thresholding-based approaches in most cases. It demonstrated how utilizing the difference of marker distribution even in a simpler form instead of a cell-specific analysis, could turn out to be more informative. In the final simulation setup, we generated datasets favoring the principles of the thresholding-based method. We showed that the JSD-based method performed competently even in that scenario.

In this article, we have focused on analyzing each of the functional markers separately. Our next goal will be to study the joint effect of multiple functional markers. One naive way of studying the joint effect would be to sum up the distance matrices corresponding to different functional markers creating a new distance matrix. This aggregated distance matrix would capture the overall difference in densities of the different markers. However, the approach is essentially assuming that the markers are independent and will be incapable of capturing complex interplay between the markers. In that light, one possible alternative would be to compare multivariate probability density of the markers across different subjects which, on the other hand, can turn out to be extremely computationally demanding. Therefore, we would study all these approaches in much greater details as a part of our next work. So far, we have tested the method on two types of multiplex imaging datasets, mIHC (Vectra) and MIBI. In future, we would like to test the applicability of our method on several other types of imaging datasets, such as HE stain (Feldman and Wolfe, 2014), newer multiplexed immunofluorescence platforms, like CODEX (Goltsev *et al.*, 2018) and MultiOmyx (Juncker-Jensen *et al.*, 2018).

## 6 Software and data avaliability

Software in the form of a GitHub *R* package, together with an example data-set and complete documentation are available at this link, https://github.com/sealx017/DenVar. The MIBI data is publicly available at https://mibi-share.ionpath.com/ and the mIHC lung cancer dataset is available upon request.

## Supplementary Material

vbac039_Supplementary_Data
