# Supplementary material for: DenVar: density-based variation analysis of multiplex imaging data
Source: Bioinform Adv. 2022 May 23;2(1):vbac039. doi: 10.1093/bioadv/vbac039 (PMC9710661; doi:10.1093/bioadv/vbac039)
Supplement: vbac039_Supplementary_Data [file vbac039_supplementary_data.pdf]

Supplementary Material for “DenVar: Density-based Variation analysis of multiplex imaging data”

Souvik Seal, Thao Vu, Tusharkanti Ghosh, Julia Wrobel and Debashis Ghosh

Department of Biostatistics and Informatics, Colorado School of Public Health,

University of Colorado Anschutz Medical Campus, Aurora, Colorado

1 TNBC dataset additional result

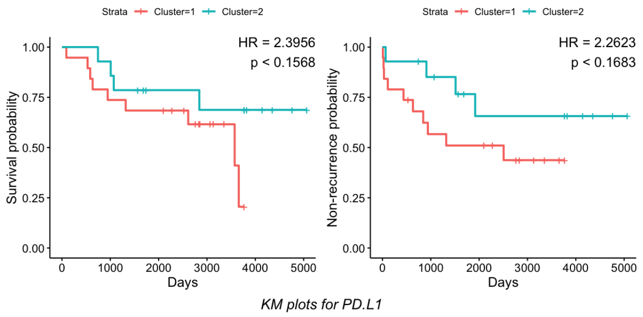

Figure S 1: KM curves for survival and recurrence probability of 33 subjects color coded by the clusters found using using our method on the marker PD-L1.

2 Details of the simulation setups (4.1) and (4.2)

In the first two simulation setups, we replicated the characteristics of the mIHC lung cancer dataset discussed in Section 3.1. In Figure (3) of the main text, we showcased the mean of HLA-DR densities of the subjects from the two clusters identified by JSD-based clustering method. We noticed that these mean densities can be well approximated using Beta distributions [1] with different sets of parameters (α, β). To find out the sets of parameters (α, β) that would approximate the mean densities of the two observed clusters, we considered the following strategy. To replicate the mean density of cluster 1, we first computed its empirical mode, denoted by m_1. We wanted to find a set of parameters, (α_1, β_1) such that Beta(α_1, β_1) satisfied two properties, a) it had the same mode, and b) its density function was close to the empirical one. To equate the modes we considered the equation, $m_1 = \frac{\alpha_1 - 1}{\alpha_1 + \beta_1 - 2}$. The value of α_1 was thus fixed for given values of β_1 and m_1. We considered different values of β_1 and chose the one for which the simulated density appeared to be closest to the real density. We repeated the above steps for replicating the mean density of cluster 2 as well.

The modes of the mean densities of clusters 1 and 2 were respectively, $m_1 = 0.0039$ and $m_2 = 0.0176$. The mean density of cluster 1 was well approximated by a Beta distribution with $\alpha_1 = 2.17, \beta_1 = 300$ and the mean density of cluster 2 by a Beta distribution with $\alpha_2 = 1.78, \beta_2 = 45$. Note that the first density had a distinctively higher peak and a narrower tail compared to the latter. Refer to Figure S1 to check how well the real and simulated densities agreed. In simulation setups (4.1) and (4.2), Beta(2.17, 300) and Beta(1.78, 45) were used respectively.

In simulation setup (4.1), two groups of subjects were considered. For a subject from the group 1, the marker expression in every cell was simulated from Beta(2.17, 300). For a subject from the group 2, the marker expression was simulated from Beta(x, 300) where x

was such that the mode of this distribution was higher than 0.0039 by a percentage of l i.e., x satisfied the equation,

$$0.0039\frac{(100+l)}{100} = \frac{x-1}{x+300-2}.$$

In simulation setup (4.2), again two groups of subjects were considered. For a subject from the group 1, the marker expression in every cell was simulated from Beta(1.78, 45). For a subject from the group 2, the marker expression was simulated from Beta(x, 45) where x was such that the mode of this distribution was higher than 0.0176 by a percentage of l i.e., x satisfied the equation,

$$0.0176\frac{(100+l)}{100} = \frac{x-1}{x+45-2}.$$

In both the setups, five different values of l, $10, 20, 50, 100$ and 200 were considered. Groups 1 and 2 respectively had sizes $N_1 = 60$ and $N_2 = 40$. Each subject had n cells and two values of n, 200 and 2000 were considered.

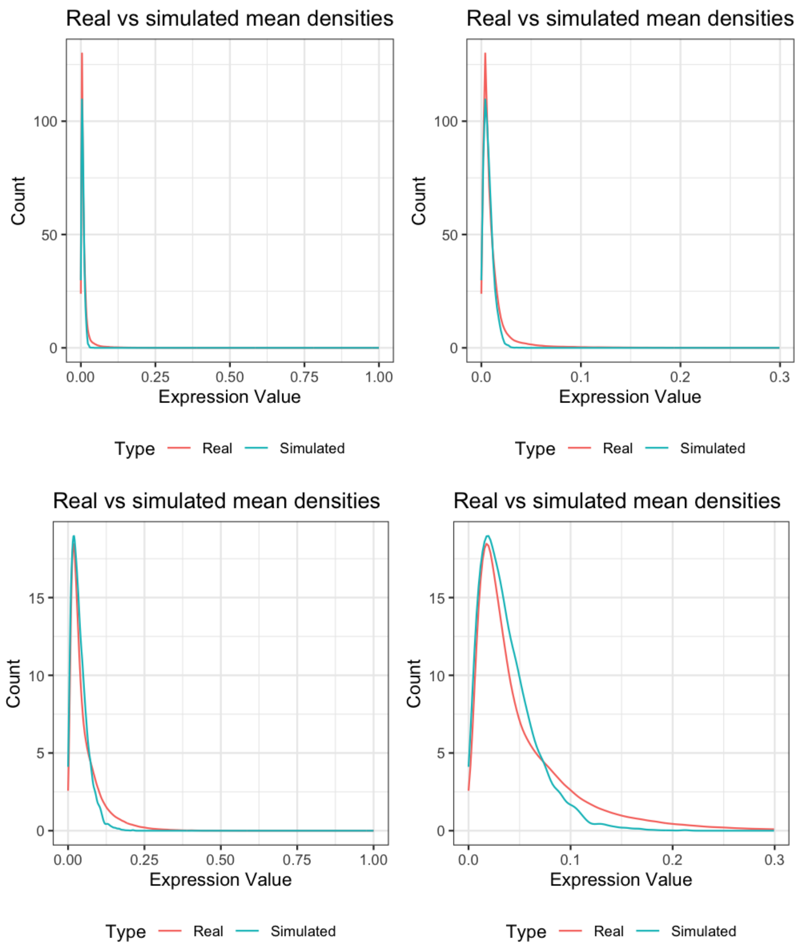

Figure S 2: The two figures on the top row show the comparison between the density of Beta$(2.17, 300)$ and the mean density of cluster 1. And, the figures on the bottom row compare the density of Beta$(1.78, 45)$ with the mean density of cluster 2. On both the rows, the figures on the left show the densities in the range, (0, 1), and the figures on the right, show the same densities in the range, (0, 0.3).

3 Details of the simulation setup (4.3)

Next, we devised a simulation where the true values of the thresholds: (t_1, t_2) were known. And the data generation process was dependent on those. Recall that t_1 controls how we define a cell to be positive for a marker and t_2 controls how we cluster the subjects into two groups. We again considered two groups with respectively $N_1 = 60$ and $N_2 = 40$ subjects each with n cells. We wanted the subjects in group 1 to have t_2% positive cells and the subjects in group 2 to have more than t_2% positive cells. We describe the process of simulating the marker data of the non-positive cells first. For subjects in group 1, we made sure that they had $(100 - t_2)$% non-positive cells by randomly choosing $n(100 - t_2)/100$ cells out of the total of n. Let $\mathcal{I}$ denote the set of indices of those non-positive cells for subject j. Next, the marker data of i-th cell from set $\mathcal{I}$, X_{ij} was simulated from Beta$(2.17, 300)$. X_{ij} can be thought of as X_{kij} from the methods section and we dropped the index k for simplicity. Once, all the X_{ij}'s were generated, the values were scaled to be in the range (0, t_1) using the transformation, $X^*_{ij} = \left(\frac{X_{ij} - min_{i \in \mathcal{I}} X_{ij}}{max_{i \in \mathcal{I}} X_{ij} - min_{i \in \mathcal{I}} X_{ij}}\right) t_1$ for $i \in \mathcal{I}$. Next, we describe the process of simulating the marker data of the positive cells. The marker data of the positive cells were again simulated from Beta$(2.17, 300)$ and a constant of t_1 was added, $X^*_{ij} = max\{X_{ij} + t_1, 1\}$, $i \in \mathcal{I}^c$. Thus, we were able to generate the whole cell-level data of a subject j from group 1, X^*_{ij} for $i \in \{1, \ldots, n\}$ making sure there were only t_2% cells having marker expression more than t_1. For subjects from group 2, a similar procedure as above was considered by replacing t_2 with a randomly chosen $t^*_2 \in (t_2, 1]$.

4 Confidence interval of ARI in simulation setups (4.1) and (4.2)

Tables S1 and S2 respectively list the confidence interval of ARI across 100 replications in the simulation setups (4.1) and (4.2).

Table S 1: Confidence interval of ARI across 100 replications for different methods in the simulation setup from Section 4.1. The intervals (0, 0) and (1, 1) respectively mean that the methods had 0 and 1 values of ARI across all replications.

Number of cells	% difference in modes	JSD based clustering	95% thresholding	97.5% thresholding	Marker quantiles
	10	(0.06, 0.0884)	(0, 0.003)	(0.018, 0.029)	(0.001, 0.009)
	20	(0.369, 0.428)	(0.001, 0.004)	(0.047, 0.064)	(0.024, 0.039)
$n = 200$	50	(0.975, 0.987)	(0.019, 0.028)	(0.209, 0.244)	(0.201, 0.239)
	100	(1, 1)	(0.177, 0.219)	(0.613, 0.652)	(0.667, 0.706)
	200	(1, 1)	(0.847, 0.879)	(0.949, 0.965)	(0.983, 0.992)
	10	(0.783, 0.822)	(0, 0)	(0, 0)	(0.063, 0.089)
	20	(0.997, 1)	(0, 0)	(0, 0)	(0.290, 0.337)
$n = 2000$	50	(1, 1)	(0, 0)	(0.002, 0.037)	(0.924, 0.946)
	100	(1, 1)	(0.002, 0.006)	(0.78, 0.84)	(0.999, 1)
	200	(1, 1)	(0.984, 0.998)	(1, 1)	(1, 1)

Table S 2: Confidence interval of ARI across 100 replications for different methods in the simulation setup from Section 4.2.

Number of cells	% difference in modes	JSD based clustering	95% thresholding	97.5% thresholding	Marker quantiles
	10	(0.025, 0.044)	(0, 0.002)	(0.011, 0.023)	(0, 0.006)
	20	(0.176, 0.225)	(0, 0.003)	(0.033, 0.049)	(0.01, 0.022)
$n = 200$	50	(0.846, 0.885)	(0.009, 0.015)	(0.127, 0.152)	(0.114, 0.148)
	100	(0.998, 1)	(0.06, 0.08)	(0.396, 0.434)	(0.420, 0.470)
	200	(1, 1)	(0.515, 0.571)	(0.856, 0.883)	(0.909, 0.930)
	10	(0.488, 0.543)	(0, 0)	(0, 0)	(0.031, 0.049)
	20	(0.967, 0.980)	(0, 0)	(0, 0)	(0.159, 0.199)
$n = 2000$	50	(1, 1)	(0, 0)	(0.002, 0.007)	(0.762, 0.797)
	100	(1, 1)	(0, 0)	(0.233, 0.292)	(0.994, 0.998)
	200	(1, 1)	(0.364, 0.45)	(0.997, 1)	(1, 1)

5 A real data example showing problems with binarizing marker expression

In Figure S2, we show five images each of the TME of two different subjects from the mIHC lung cancer dataset. In every image, the HLA-DR marker expression is plotted across the cells at their xy co-ordinates. Notice that in all the images from subject 2, the marker expression is mostly close to zero, whereas in some of the images from subject 1, the marker expression is noticeably high. Thus, these two subjects should ideally be distinguishable if

the difference is quantified in terms of their overall marker expression.

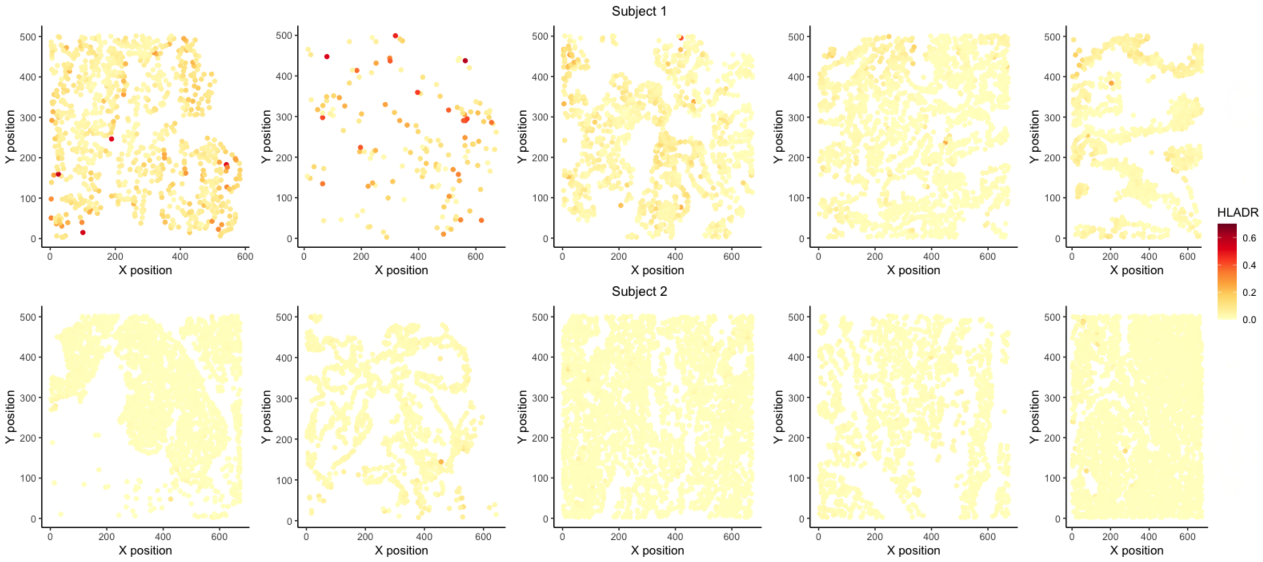

Figure S 3: HLA-DR marker expression in the cells of the TME of two subjects from the mIHC lung cancer dataset. Five images on the top row correspond to subject 1 and the bottom row corresponds to subject 2. A deeper color indicates higher expression value.

In Figure S3, we binarize these images meaning that we declare a cell to have marker expression one or zero based on its true expression being more or less than a chosen threshold of 0.55. We observe that subject 1 now has mostly zero marker expression bar just two cells and thus, becomes nearly indistinguishable from subject 2. This was probably the reason why subjects 1 and 2 were both assigned to be in MHC-II low group by the thresholding-based method, whereas our method could identify their differences and assigned them into separate clusters. This example shows how discarding important marker information (by binarizing) can potentially result in an overall loss of power.

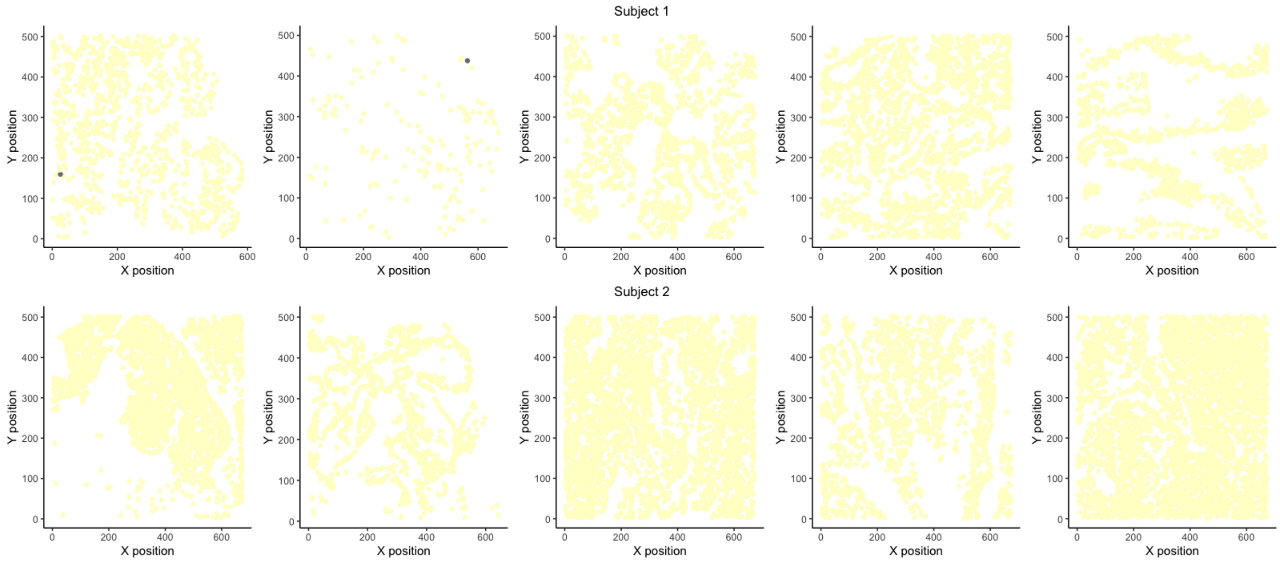

Figure S 4: Binarized marker expression in the images of the two subjects at a threshold of 0.55.

References

[1] Arjun K Gupta and Saralees Nadarajah. *Handbook of beta distribution and its applications.* CRC press, 2004.
